# Underrecognition and undertreatment of thirst among hospitalized patients with restricted oral feeding and drinking

**DOI:** 10.1038/s41598-021-93048-4

**Published:** 2021-07-01

**Authors:** Vanda Ho, Gordon Goh, Xuan Rong Tang, Kay Choong See

**Affiliations:** 1 Department of Geriatric Medicine, National University Hospital, 5 Lower Kent Ridge Rd, Singapore, 119074 Singapore; 2 Yong Loo Lin School of Medicine, National University, Singapore, Singapore; 3 Division of Respiratory and Critical Care Medicine, Department of Medicine, National University Hospital, Singapore, Singapore

**Keywords:** Nutrition disorders, Quality of life, Signs and symptoms

## Abstract

Thirst is distressing but overlooked by healthcare professionals. Patients experience thirst due to comorbidities, physical or cognitive limitations, and iatrogenesis. Nasogastric tube (NGT) use and nil-by-mouth(NBM) orders are common practices that can lead to thirst. However, thirst in these populations has never been formally studied. We aim to examine prevalence of recognition and treatment of thirst among NGT + NBM and NBM patients. Our descriptive study was conducted intermittently over 25 weeks, across 1.5 years, in 12 adult general medicine wards of a tertiary hospital. Cognitively intact NGT + NBM or NBM inpatients, defined as Abbreviated Mental Test score ≥ 8, were studied. One-time questionnaire was administered. Variables included: demography, co-morbidities, clinical condition, feeding route, thirst defined by thirst distress and/or intensity ≥ 3, pain, hunger and volume status. 88 NGT + NBM and NBM patients were studied. 69.3% suffered from thirst. Thirsty patients experienced significant thirst-related distress (mean score ± SD: 5.7 ± 2.5). Subjects with previous stroke and who were euvolemic tended towards thirst. 13.6% were asked about thirst by doctors or nurses. Thirst was a major source of patient distress in our study. We suggest that thirst needs to be actively identified and targeted to achieve person-centred care.

## Introduction

Thirst is defined as the “sensation of dryness in the mouth and throat associated with a desire for liquids”^1^. It is critical in the maintenance of serum osmolarity and cellular homeostasis. Patients experience thirst for many reasons. Physiologically, increased serum osmolarity or depleted intracellular volume leads to release of anti-diuretic hormone (ADH). Over time, activation of renin-aldosterone-angiotensin system (RAAS) causes angiotensin-II release which acts on the hypothalamus to stimulate thirst^2^. Problems arise when there are exacerbating factors, such as medical or mental comorbidities, physical or cognitive limitations, medications or peri-procedural restrictions^3^.

Prevalence of thirst ranges from 43.8 up to 90% in intensive care unit (ICU) patients, people who are on strict fluid balance such as those with heart failure and haemodialysis, and patients receiving palliative care^4–6^. The appropriate response to thirst would be to obtain and ingest fluids. However, these may be challenging in those who are less mobile, have restricted access to water or on medications which worsen thirst. In ICU patients who often fall into all three categories, Puntillo and colleagues have utilised low-cost effective interventions of oral swab wipes, ice water sprays and lip moisturiser^7^. There was marked reduction in thirst and improvement in these patients’ quality of life.

Despite its prevalence, thirst is commonly overlooked by healthcare professionals, which can be attributed to a lack of clinician awareness, lack of systemic identification of thirst during a clinical encounter and lack of evidence-based management^8^. When unaddressed, thirst can present as a distressing symptom comparable to pain^3^. Much like how pain has been established to be our “fifth vital sign”^9^, we believe that detecting and treating thirst have equal importance. Being able to detect thirst can also help to alleviate caregiver stress and the emotional distress of patients^5^.

A population vulnerable to thirst but has been less studied are people on nasogastric tubes (NGT). Padilla and colleagues found thirst to be the most distressing symptom in those on NGT. The reasons are multifactorial. They are often kept nil-by-mouth (NBM) and if otherwise, often have limited mobility which may restrict their access to oral fluids and further perpetuate their thirst distress. They often breathe through their mouths and dry mouths can cause significant discomfort^10^. In terms of intervention, there have not been any studies looking into NGT patients. The high risk of aspiration in people on NGT^11^ poses as a barrier. This risk often makes their loved ones and the clinical team uneasy about providing measures for thirst due to fear of causing harm. Nonetheless, the positive impact on thirst and lack of increased aspiration risk from Puntillo’s ICU study are assuring, and we feel that that patients on NGT needs to be studied closer for implementation of safe cost-effective interventions for improving quality of life.

We hypothesize potential under-recognition and under-treatment in patients with NGT and who are kept NBM, and we aim to study this. Our results can inform the need for quality improvement to alleviate thirst among these patients.

## Methods

Our descriptive study was conducted intermittently over 25 weeks, from August 2018 to February 2020. The study was conducted at the National University Hospital, a 1200-bed public teaching hospital with a full range of medical and surgical specialties. Local ethics approval was obtained (reference number: 2018/00371). Informed consent was obtained from all participants.

Study investigators screened patients on NGT and NBM (NGT + NBM) and who were NBM from all 12 general internal medicine wards. Due to the prolonged length of stay of NGT patients, the wards were sampled at approximately 3-weekly intervals to ensure the same patients were not interviewed. Inclusion criteria included inpatients at least 21 years of age; conversant in English or Mandarin; deemed appropriate by primary healthcare team; abbreviated mental test (AMT) score of at least 8 and above; on NGT feeding or NBM; consent to participate in this study. NGT patients interviewed were also NBM, and patients in the NBM group did not have NGTs. Patients who were unable to communicate either verbally or in writing were excluded.

Members of the study team underwent standardised training prior to conducting interviews. A fixed set of instructions were given to team members on how to approach potential subjects, measures to ensure privacy, acceptable explanations of terms and evaluation of volume status. Information gathered from the interview included: AMT score, patient demographic and co-morbidities, current clinical condition, feeding route, thirst, scores for thirst intensity (TI) and distress (TD) respectively (Appendix 1). Thirst has been quantified on an 11-point Numerical Rating Scale (NRS), from 0 to 10 (10 being the most intense or distressing). This scale has been well-established in assessing pain intensity^12,13^, and has been used in evaluating thirst in patients with congestive cardiac failure and haemodialysis^14–16^. Use of the thirst NRS can also be conducted in patients who are not able to communicate verbally or mildly cognitive impaired^17,18^, as is often the case in those who are on NGT feeding. Thirst is defined as thirst distress and/or intensity 3 or more^7^. Pain and hunger scores were obtained as confounders of thirst. Interviewers also performed a brief bedside evaluation of displayed feeding signs, ease of access to water and a clinical evaluation on the patient’s volume status. Objective signs include peripheral oedema, lung crepitations, ascites for hypervolemia and sunken orbits, reduced skin turgor and dry mucosa for hypovolemia. There are no prior studies examining visually ascertained volume status in NGT/ NBM patients. We have extrapolated results from studies done in older adults in the emergency department, where they found dry mucous membranes, dry tongue, tongue furrows, sunken eyes to have sensitivity ranging 59 to 85% and specificity ranging 58 to 82% to the ground truth of elevated serum urea nitrogen-creatinine ratio, serum osmolality and sodium^19^. Another study looked at patients who are vomiting, diarrhoea or have decreased oral intake, and found dry axilla to support hypovolemia with a positive likelihood ratio of 2.8, while moist mucous membranes and a tongue without furrows argued against it^20^.

Stata (Version 14.0) was used for statistical analysis. Descriptive statistics were performed. Student’s T tests and Fisher’s exact test were carried out to compare differences in demographic, clinical and nursing indices between the NGT + NBM and NBM groups. For non-parametric data, Wilcoxon rank sum test was used. All date was described as mean ± standard deviation (SD) or median (inter quartile range; IQR). Results are presented to 1 decimal place. A *P* value of < 0.05 was considered statistically significant.

This study was approved by the National Healthcare Group (NHG) ethics committee (domain specific review board reference number: 2018/00371). All methods were performed in accordance with the relevant guidelines and regulations.

### Ethics approval

Approved by the National Healthcare Group (NHG) ethics committee (domain specific review board reference number: 2018/00371).

## Results

**Figure 1:**
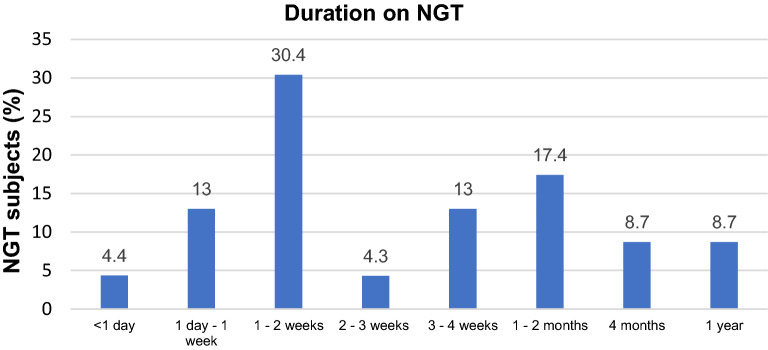
Graph of NGT + NBM subjects by duration on NGT.

88 subjects on NGT + NBM (n = 57) or NBM (n = 31) were interviewed. Mean age of participants was 64.3 ± 14.9 years, 67.0% were males. 80.7% were Chinese. Mean number of comorbidities per participant was 1.67, of which the most common were hypertension (54.5%) and cancer (40.9%). NGT + NBM subjects tended to have more cancer and stroke than those on NBM. The most common reason for admission was gastrointestinal issues (44.3%). The NBM group had more admissions for infections beyond a urinary or respiratory source. The most common medications given were intravenous fluids (77.3%) and proton pump inhibitors (69.3%). Median length of stay in hospital was 5 (2–15) days when interviewed, with 29.5% transferred from an ICU or a high dependency unit (HD) to the general floor. Subjects in the NGT + NBM group had stayed in hospital longer and tended to need ICU or HDU care. NGT + NBM subjects spent more hours without oral intake at time of interview (median for NGT + NBM 336 (168–1440) h versus NBM 12 (8–17) h, *P* < 0.001). The largest proportion of NGT + NBM subjects (30.4%) were on NGT for 1 to 2 weeks, as seen in Fig. 1. The main indications for NGT + NBM and NBM were for intervention. Clinical characteristics of NGT + NBM versus NBM are detailed in Table 1.

**Table 1:** Clinical Characteristics between the NGT + NBM and NBM groups.

Characteristics	All, n = 88 (100%)	NGT + NBM, n = 57 (64.8%)	NBM, n = 31 (35.2%)	*P* value
**Age**				
Mean ± SD^a^	64.3 ± 14.9	65.3 ± 12.7	62.5 ± 18.5	0.4
**Sex**				
Male (n, %)	59 (67.0)	35 (61.4)	24 (77.4)	0.2
**Race**				
Chinese (n, %)	71 (80.7)	47 (82.5)	24 (77.4)	0.3
Malay (n, %)	5 (5.7)	2 (3.5)	3 (9.7)	
Indian (n, %)	11 (12.5)	8 (14.0)	3 (9.7)	
Others (n, %)	1 (1.1)	0 (0.0)	1 (3.2)	
**Comorbidities**				
Hypertension (n, %)	48 (54.5)	31 (54.4)	7 (22.6)	1.0
Cancer (n, %)	36 (40.9)	29 (50.9)	7 (22.6)	0.01*
Diabetes mellitus (n, %)	34 (38.6)	23 (40.4)	11 (35.5)	0.8
Stroke (n, %)	16 (18.2)	14 (24.6)	2 (6.5)	0.04*
Ischemic heart disease (n, %)	11 (12.5)	5 (8.8)	6 (19.4)	0.2
Chronic kidney disease (n, %)	5 (5.7)	2 (3.5)	3 (9.7)	0.3
Chronic pulmonary disease (n, %)	5 (5.7)	3 (5.3)	2 (6.5)	1.0
Depression (n, %)	2 (2.3)	1 (1.8)	1 (3.2)	1.0
**Reason for admission**				
Gastrointestinal (n, %)	39 (44.3)	26 (45.6)	13 (41.9)	0.8
Infection (not respiratory/urinary) (n, %)	15 (17.0)	5 (8.8)	10 (32.3)	0.008*
Trauma (n, %)	8 (9.1)	4 (7.0)	4 (12.9)	0.4
Respiratory (n, %)	5 (5.7)	5 (8.8)	0 (0.0)	0.2
Cardiovascular (n, %)	4 (4.6)	1 (1.8)	3 (9.7)	0.1
Urinary (n, %)	4 (4.6)	2 (3.5)	2 (6.5)	0.6
Neurological (n, %)	3 (3.4)	3 (5.3)	0 (0.0)	0.5
Hematological (n, %)	3 (3.4)	2 (3.5)	1 (3.2)	1.0
Metabolic (n, %)	3 (3.4)	2 (3.5)	1 (3.2)	1.0
Elective surgery (n, %)	3 (3.4)	3 (5.3)	0 (0.0)	0.5
Head and neck lumps (n, %)	2 (2.3)	2 (3.5)	0 (0.0)	0.5
**Medications**				
Intravenous fluids (n, %)	68 (77.3)	43 (75.4)	25 (80.6)	0.8
PPIs^b^ (n, %)	61 (69.3)	39 (68.4)	22 (71.0)	1.0
Opioids (n, %)	40 (45.5)	28 (49.1)	12 (38.7)	0.4
Anti hypertensives (n, %)	35 (39.8)	20 (35.1)	15 (48.4)	0.3
Corticosteroids (n, %)	9 (10.2)	5 (8.8)	4 (12.9)	0.7
NSAIDS^c^ (n, %)	18 (20.5)	8 (14.0)	10 (32.3)	0.06
Diuretics (n, %)	10 (11.4)	7 (12.3)	3 (9.7)	1.0
Tricyclic antidepressants (n, %)	2 (2.3)	2 (3.5)	0 (0.0)	0.5
Anticholinergics (n, %)	2 (2.3)	2 (3.5)	0 (0.0)	0.5
Antipsychotics (n, %)	1 (1.1)	1 (1.8)	0 (0.0)	1.0
**Hospital admission**				
Median (IQR)^d^ (days)	5 (2–15)	9.5 (3.5–23)	2 (1–5)	< 0.001*
**Intensive care/high dependency unit during the admission**				
Yes (n, %)	26 (29.5)	25 (43.9)	1 (3.2)	< 0.001*
**NGT/NBM**				
Median (IQR)^d^ (hours)	24 (11–264)	336 (168–1440)	12 (8–17)	< 0.001*
NGT before current admission Yes (n, %)	7 (8.0)	5 (8.8)	2 (6.5)	1.0
**Reason for NGT/NBM**				
Surgery/intervention (n, %)	63 (71.6)	37 (64.9)	26 (83.9)	0.08
Swallowing impairment (n, %)	13 (14.8)	13 (22.8)	0 (0.0)	0.003*
Poor oral intake (n, %)	10 (11.4)	10 (17.5)	0 (0.0)	0.01*
**Thirst indices**				
Thirst present (n, %)	61 (69.3)	43 (75.4)	18 (58.1)	0.1
Thirst distress score^+^, mean ± SD^a^	4.0 ± 3.3	4.4 ± 3.4	3.2 ± 3.1	0.08
Thirst intensity score^+^, mean ± SD^a^	4.2 ± 3.6	4.7 ± 3.6	3.3 ± 3.4	0.7
Hunger score^+^, mean ± SD^a^	2.9 ± 3.0	2.6 ± 3.1	3.3 ± 2.9	0.6
Pain score^+^, Mean ± SD^a^	1.7 ± 3.0	1.6 ± 3.0	2.0 ± 8.8	0.4

*P < 0.05 for comparison between NGT and NBM groups.

^a^SD: Standard deviation.

^b^PPI: Proton Pump Inhibitor.

^c^NSAID:Non-steroidal anti-inflammatory drugs.

^d^IQR: inter-quartile range.

+ scored 0 to 10, 10 being most severe.

69.3% (n = 61) reported thirst. TD and TI correlated positively (R = 0.9, *P* < 0.01). Indices comparing NGT + NBM versus NBM are described in Table 1. 75.4% NGT + NBM subjects experienced thirst, compared to 58.1% of those who were NBM, though the difference is not statistically significant (*P* = 0.1).

Indices comparing thirsty versus not thirsty are described in Table 2. Those who reported thirst had greater TD and TI scores. Pain and hunger scores were not associated with higher thirst indices.

**Table 2:** Predictors of Thirst.

Characteristics	All, n = 88 (100.0%)	Thirsty, n = 61 (69.3%)	Not thirsty, n = 27 (30.7%)	*P* value
**Age**				
Mean ± SD^a^	64.3 ± 14.9	64.9 ± 13.4	63.0 ± 18.0	0.6
**Gender**				
Male (n, %)	59 (67.0)	40 (65.6)	19 (70.4)	0.8
**Race**				
Chinese (n, %)	71 (80.7)	50 (82.0)	21 (77.8)	0.8
Malay (n, %)	5 (5.7)	3 (4.9)	2 (7.4)	
Indian (n, %)	11 (12.5)	7 (11.5)	4 (14.8)	
Others (n, %)	1 (1.1)	1 (1.6)	0 (0.0)	
**Comorbidities**				
Hypertension (n, %)	48 (54.5)	35 (57.4)	13 (48.1)	0.5
Cancer (n, %)	36 (40.9)	26 (42.6)	10 (37.0)	0.6
Diabetes mellitus (n, %)	34 (38.6)	28 (45.9)	6 (22.2)	0.06
Old cerebrovascular accident (n, %)	16 (18.2)	15 (24.6)	1 (3.7)	0.02*
Ischemic heart disease (n, %)	11 (12.5)	8 (13.1)	3 (11.1)	1.0
Chronic kidney Disease (n,%)	5 (5.7)	4 (6.6)	1 (3.7)	1.0
Chronic pulmonary disease (n, %)	5 (5.7)	4 (6.6)	1 (3.7)	1.0
Depression (n, %)	2 (2.3)	0 (0.0)	2 (7.4)	0.09
**Reason for admission**				
Gastrointestinal (n, %)	39 (44.3)	28 (45.9)	12 (44.4)	0.8
Infection (not respiratory/urinary) (n, %)	15 (17.0)	10 (16.4)	5 (18.5)	0.8
Trauma (n, %)	8 (9.1)	4 (6.6)	4 (14.8)	0.2
Respiratory (n, %)	5 (5.7)	4 (6.6)	1 (3.7)	1.0
Cardiovascular (n, %)	4 (4.6)	4 (6.6)	0 (0.0)	0.3
Urinary (n, %)	4 (4.6)	3 (4.9)	1 (3.7)	1.0
Neurological (n, %)	3 (3.4)	1 (1.6)	2 (7.4)	0.2
Hematological (n, %)	3 (3.4)	3 (4.9)	0 (0)	0.6
Metabolic (n, %)	3 (3.4)	2 (3.3)	1 (3.7)	1.0
Elective surgery (n, %)	3 (3.4)	3 (4.9)	0 (0.0)	0.6
Head and neck lumps (n, %)	2 (2.3)	0 (0.0)	2 (7.4)	0.09
**Medications**				
Intravenous fluids (n, %)	68 (77.3)	50 (82.0)	18 (66.7)	0.2
PPIs^b^ (n, %)	61 (69.3)	43 (70.5)	18 (66.7)	0.8
Opioids (n, %)	40 (45.5)	31 (50.8)	9 (33.3)	0.2
Anti hypertensives (n, %)	35 (39.8)	25 (41.0)	10 (37.0)	0.8
Corticosteroids (n, %)	9 (10.2)	8 (13.1)	1 (3.7)	0.3
NSAIDS^c^ (n, %)	18 (20.5)	12 (26.4)	6 (22.2)	0.8
Diuretics (n, %)	10 (11.4)	9 (14.8)	1 (3.7)	0.2
Tricyclic antidepressants (n, %)	2 (2.3)	1 (1.6)	1 (3.7)	0.5
Anticholinergics (n, %)	2 (2.3)	2 (3.2)	0 (0.0)	1.0
Antipsychotics (n, %)	1 (1.1)	1 (1.6)	0 (0.0)	1.0
**Duration of hospital**				
Median (IQR)^d^ (days)	5 (2–15)	4 (1–10)	6 (3–17)	0.07
**ICU/HDU this admission**				
No (n, %)	62 (70.5)	42 (68.9)	20 (74.1)	0.8
**NGT or NBM**				
NGT (n, %)	57 (64.8)	43 (70.5)	14 (51.9)	0.1
**Duration on NGT/NBM** Median (IQR)^d^ (h)	24 (11–264)	36 (10–336)	20.5 (12–252)	1.0
**NGT before current admission**				
No (n, %)	81 (92.0)	56 (91.8)	25 (92.6)	1.0
**Volume status**				
Hypervolemic (n, %)	1 (1.1)	0 (0.0)	1 (3.7)	0.3
Hypovolemic (n, %)	41 (46.6)	24 (39.3)	17 (63.0)	0.06
Euvolemic (n, %)	46 (52.3)	37 (60.7)	9 (33.3)	0.02*
**Doctors/nurses asked about thirst**				
No (n, %)	76 (86.4)	50 (82.0)	26 (96.2)	0.1
**Doctors/nurses attempted to treat thirst**				
No (n, %)	65 (73.9)	42 (68.9)	23 (85.2)	0.1
**Thirst indices**				
Thirst distress score^+^, mean ± SD^a^	4.0 ± 3.3	5.7 ± 2.5	0.1 ± 0.4	< 0.001*
Thirst intensity score^+^, mean ± SD^a^	4.2 ± 3.6	6.1 ± 2.7	0.0 ± 0.2	< 0.001*
Hunger score^+^, mean ± SD^a^	2.9 ± 3.0	3.3 ± 3.0	2.0 ± 2.9	0.3
Pain score^+^, Mean ± SD^a^	1.7 ± 3.0	1.5 ± 2.7	2.2 ± 3.5	0.08

*P < 0.05.

^a^SD: Standard deviation.

^b^PPI: Proton Pump Inhibitor.

^c^NSAID:Non-steroidal anti-inflammatory drugs.

^d^IQR: inter-quartile range.

^+^scored 0 to 10, 10 being most severe.

Predictors of thirst are described in Table 2. Patients with previous stroke tended to report feeling thirsty. More subjects with diabetes were thirsty, though this was not significant. Age, treatment administered during the hospital admission, duration or indication for NGT or NBM did not affect thirst indices. Those who were thirsty tended to be euvolemic. Euvolemic NGT + NBM subjects had higher TI scores compared to hypovolemic NGT + NBM subjects (hypovolemic: mean score 3.3 ± 3.7, versus euvolemic: 5.9 ± 3.2, *P* = 0.02), this relationship was not seen in the NBM group. 13.6% subjects were asked about thirst by doctors or nurses, and those who were thirsty reported being asked less. Of methods used to treat thirst, allowing sips of water was used most, followed by ice chips, but these were not associated with lower thirst indices.

## Discussion

Prevalence of thirst was high at 69.3%. Subjects who were thirsty also experienced significant distress related to thirst. Both hunger and pain were not associated. Subjects with previous stroke and those who were euvolemic tended towards thirst. Only 13.6% were asked about thirst, though no treatment modality was associated with lower thirst scores.

Our study found a high thirst prevalence of 69.3% in NGT + NBM and NBM patients. Thirst has not been previously explored in general ward NGT or NBM patients. Specific patient populations such as palliative and ICU patients have been examined, with high thirst prevalence of up to 90%^6^ and 70%^21^ respectively. These patients often were on NGT or NBM, though generalisability to our population is difficult as these patients tended to be sicker. This difference might account for the slightly lower prevalence seen in our population. As NGT feeding is the most common form of artificial feeding inpatient^22^, even with our slightly lower thirst prevalence, this problem still affects a large number of patients. Additionally, up to 50% of long-term care residents are on long-term NGT^23^, suggesting that thirst has far-reaching implications. Previous studies found pre-operative patients to be more bothered by the discomfort caused by thirst than that of not being to eat or sleep^24^. Hence, with our knowledge of high thirst prevalence, there is a call for more work to be done to identify and to treat thirst.

Our study suggest that previous stroke can be a contributor to thirst. One plausible reason is that the cerebral insult may affect sensory circumventricular organs or their connection to the cortex. This neural network controls thirst^25^, and damage to the pathway can cause thirst dysregulation.

There were no contributors of thirst pertaining specifically to the NGT + NBM or NBM group. This differs to previous contributing factors seen in specific populations. For example, in ICU patients, high opioid doses and furosemide doses, selective serotonin reuptake inhibitors and low ionized calcium were determined to predict thirst^3^. Opioids and diuretics were not found to be associated in our population, though the low rates of medication use may account for this difference. For our population, thirst contributors were different due to their unique set of comorbidities. Interestingly, while those with NGT + NBM had more severe physical conditions including dysphagia, there were no significant differences found in thirst indices. We expected that those with NGT to experience more subjective thirst distress^10^, due to reasons such as preferentially breathing through their mouths secondary to discomfort from the tube, and limited mobility restricting access to thirst relieving measures such as ice chips and lip moisturisers. A potential explanation is that those on NGT + NBM had been in this state for longer (median of 336 h compared to 12 h for our NBM group) and have adapted over time with resetting of their thirst osmostat. Unexpectedly, age did not correlate with thirst in our study. These patients might have a blunted response to thirst due to age or disease-mediated changes on thirst receptors and perception.

The euvolemic NGT + NBM patients experienced more thirst intensity and this might have triggered them to ask for water more proactively. Our study focussed on bedside evaluation of thirst and we do not have information on objective laboratory markers of dehydration such as serum sodium and osmolarity. Despite clinical euvolaemia, it is possible that these patients may be hyperosmolar or hypernatremic and this may make them feel thirsty. As thirst can be driven by either intracellular or extracellular dehydration, or both, clinically assessed volume status may not reflect serum osmolality^26^. This would warrant further investigation. Furthermore, there is a possibility that these patients were given IV fluids to treat hyperosmolarity, hypernatremia or hypovolemia, which corrected the fluid balance without eliminating the thirst sensation. Sensation of thirst is multifactorial. Sources of input includes signals from the gut on ingested osmolarity^27^ and detection of water in the oropharynx. Hence, without the oropharynx stimulation, thirst may persist^26,28^. Therefore, specific populations have to be examined to identify their risk factors.

Thirst was under-recognized. Majority of patients were not asked about thirst by members of their primary care team. Worryingly, these patients tended to have more thirst distress. Our study detected thirst using simple tools of direct questioning and NRS for severity, which had been used prior^14–16^. It is possible that the healthcare team recognises thirst by laboratory signs of dehydration such as serum osmolarity, sodium and hemocrit, rather than patients’ report. However, thirst is also heavily affected by oral sensation, which is unaddressed with IV fluids. The team’s efforts of ameliorating thirst such as giving IV fluids may thus not be appreciated by patients. This is seen from our finding that a greater fraction of thirsty patients received IV fluids (82% vs. 67%), which may suggest physicians’ recognition and treatment of fluid imbalance. Hence, communication between patient and healthcare team has to be enhanced. As a way forward, this tool can be implemented on a wider scale to characterise thirst in different populations.

Thirst was also under-treated. In our current hospital setting, treatment is patient-triggered. This would be a problem for many NGT patients who are cognitively impaired or have concomitant communicative deficits. Ice chips and sips of water were the predominant methods of treating thirst, and had been found to be effective in patients in heart failure^5^. In terms of effective intervention, education is first needed^29^. For tube feeding, the complications usually cited are that of pulmonary, gastrointestinal or local trauma^30^. The patient experience is often forgotten, and indeed patients should be counselled on the common and distressful experiences of sensory irritation and deprivation^10^ prior to insertion. Importantly, perception of thirst does not appear to be mediated by administering of IV fluids.

Our study was predominantly patient-centred, and doctors were not approached to check their understanding or attempt at treating thirst. It could be possible that doctors recognised thirst and attempted to treat them with IV fluids to correct the physiological derangement such as hemotocrit or osmolarity. However, thirst can persist even after circulatory signs have been corrected as seen in studies in humans^28^ and mice^27^, and can be due to potent oral sensations like mouth dryness producing a sense of thirst. This highlights the complex nature of thirst beyond physiology and the need for holistic assessment of the person.

### Strengths and limitations

Our study’s strength lies in examining a ubiquitous high-risk population which had not been characterised previously. We identified NGT and NBM patients as most in need of symptom identification and intervention. We intentionally did not focus on a singular medical condition to allow broad application of our study results for the hospitalist. Comparing between the NGT and NBM had allowed us to identify the variation in thirst contributors and manifestation.

Our recruitment was slower than expected as many NGT patients were cognitively impaired. Furthermore, the turnover for NGT patient admissions was not high. Some patients might have difficulty understanding the difference in distress and intensity. We mediated this potential inconsistency by mandating all raters to undergo a standardised training prior to data collection. Raters were trained to have a fixed spiel to explain the difference if prompted. To minimise variability in the visual determination of volume status, raters underwent training prior to starting data collection. We did not collect quantitative markers of hydration status such as serum osmolality, sodium and hematocrit, and this is warrants further study.

Our study did not test for multiple comparisons as we may have false positive results. Using the Bonferroni-corrected *P*-value of 0.05/42 = 0.001, none of the predictors are statistically significant. Hence, our study is hypothesis-generating, and more studies with either larger sample sizes or having a more targeted approach to predictor-identification would be required to confirm our preliminary results.

### Clinical implications and future work

Our study has important implications for daily hospital practice. We show that thirst is under-detected, and this can be rectified with systematic use of direct questioning and NRS scales. These tools are quick and simple, making it potentially feasible for regular clinical evaluation of thirst. A potential additional parameter that can be explored and has been found to correlate with thirst is that of tachycardia^31^. As parameter monitoring is already routine inpatient, heart rate can contribute to an objective measure of thirst. We also identify risk factors to highlight to the clinician to evaluate further, such as diabetes, stroke and cardiovascular conditions. We hope for further studies in other patient populations, as well as development of cost-effective strategies to alleviate thirst. Current literature shows conflicting results on chewing gum and saliva substitute^32–34^. Puntillo and team’s ICU nursing thirst bundle including low cost intervention of oral swab wipes, sterile ice-cold water sprays and lip moisturiser^7^ shows promise and has potential for replication on the general ward.

## Conclusion

We believe our study is a start into looking at inpatient thirst. We have shown that thirst is highly prevalent in NGT and NBM patients. Thirst is an independent major source of patient distress which needs to be identified and targeted in our goal towards person-centred care. Previous stroke is linked to thirst. Those with normal volume status complained about thirst more than in those who had abnormally high or low status. Further studies about correlation between subjective thirst and objective quantitative assessments or laboratory markers such as serum osmolarity, sodium and hematocrit, are needed. Only 13.6% were asked about thirst, which is in contrast to more than half of these patients receiving IV fluids. Hence, whilst the healthcare team might be treating thirst, the lack of communication with patients and lack of awareness of the importance of potent oral sensation for thirst satiation have led to patients feeling that their thirst is under-recognized. Therefore, clinician and nursing awareness need to be raised, and improved communication to patients is critical. Patients should be actively asked about thirst and what it means to them, especially in the presence of high-yield interventions. We hope that future studies can be done in other populations, such as home care and elderly, with plans for low-cost intervention to be examined subsequently to improve quality of life for our patients.

## Supplementary Information

Supplementary Information 1.

Supplementary Information 2.

Supplementary Information 3.

Supplementary Information 4.
