# Supplementary material for: Underrecognition and undertreatment of thirst among hospitalized patients with restricted oral feeding and drinking
Source: Sci Rep. 2021 Jul 1;11:13636. doi: 10.1038/s41598-021-93048-4 (PMC8249500; doi:10.1038/s41598-021-93048-4)
Supplement: Supplementary file 2 — Supplementary Information 2. [file 41598_2021_93048_MOESM2_ESM.docx]

# Title page

**Title**: Under-recognition and under-treatment of thirst among general floor inpatients requiring nasogastric tubes and who are kept nil-by-mouth: an observational study

**Authors**

*Vanda Ho^1^ MB Chir, MRCP (UK)

Gordon Goh^2^

Xuan Rong Tang^2^

Kay Choong See^3^ MBBS, MRCP(UK), FRCP Edin, MPH(Harvard), MHPE(Maastricht), EDIC, FCCP

^1^ Department of Geriatric Medicine, National University Hospital Singapore

^2^Yong Loo Lin School of Medicine, National University Singapore

^3^ Division of Respiratory and Critical Care Medicine, Department of Medicine, National University Hospital, Singapore

e0238044@u.nus.edu

[xuanrong.tang@u.nus.edu](mailto:xuanrong.tang@u.nus.edu)

kay_choong_see@nuhs.edu.sg

**Corresponding Author**

Vanda Ho

E-mail address: [vanda_wt_ho@nuhs.edu.sg](mailto:vanda_wt_ho@nuhs.edu.sg)

Mailing address: National University Hospital, 5 Lower Kent Ridge Rd, Singapore 119074

+65-6779 5555

**Word count**: 2814

Number of tables: 4

Number of figures: 2

Supplementary Material: Appendix 1

**Key words:** thirst, NGT, NBM, inpatient

# Supplementary Material

Appendix 1: Questionnaire for Study on Thirst in General Ward Patients on Nasogastric Tube Feeding/ Nil-by-mouth

Patient demographics

- Age
- Gender
  - Male
  - Female
- Race
  - Chinese
  - Malay
  - Indian
  - Eurasian
  - Others
- Language used to conduct interview
  - English
  - Chinese
  - Malay
  - Tamil
  - Others

Co-morbidities

- Depression, HTN, DM, IHD, asthma, COPD, CKD, chronic liver disease, old stroke, cancer

Current clinical condition

- Why did you come to hospital?
  - Stroke
  - Heart attack
  - Pneumonia
  - Urinary tract infection
  - Asthma
  - COPD
  - BGIT
  - Renal failure
  - Others, state: _____
- What treatment have you had so far?
  - Opioids
  - Anticholinergics
  - Diuretics
  - Tricyclic antidepressants
  - SSRIs
  - Nonsteroidal anti-inflammatory drugs
  - Corticosteroids
  - Proton pump inhibitors
  - Antihypertensives
  - IV fluids
- How long have you been in hospital for? (day of enrolment)
- Were you admitted to ICU/HD? Y/N
  - If yes, how long were you intubated for? ___ days / NA (never intubated)

Feeding route

- Were you on NGT before this admission?
  - If yes, how long have you been on NGT for?
- Why was NGT inserted?
  - Swallowing impairment
  - Surgery/ intervention
  - Poor oral intake
- Are you allowed to take anything orally?
  - Is there water by bedside?
  - Is it within reach?
- Is there a feeding sign above patient’s bed? What does it say?
  - None
  - Aspiration risk
  - NBM
  - Allow sips of water

Thirst

- Are you thirst? Y/N
- How distressing or bothersome is your thirst? 0 = no distress, 10 = very distressing
- How intense is your thirst? 0 = no thirst, 10 = worse possible thirst
- Did any doctor or nurse ask you about thirst? Y / N
  - If yes: Did any doctor or nurse attempt to treat your thirst? Y / N
    - If yes, how did any doctor or nurse treat your thirst? Wet lips / allow sips of water / ice chips/ other ___ (text)
- How hungry are you? 0 = no hunger, 10 = worst possible hunger
- How much pain do you have? 0 = no pain, 10 = worst possible pain

Observed: Can I check your mouth and skin? (Check oral mucosa and skin turgor)

- Hydration status (observed)
  - Hypovolaemic
  - Euvolaemic
  - Hypervolaemic
