# Supplementary material for: Underrecognition and undertreatment of thirst among hospitalized patients with restricted oral feeding and drinking
Source: Sci Rep. 2021 Jul 1;11:13636. doi: 10.1038/s41598-021-93048-4 (PMC8249500; doi:10.1038/s41598-021-93048-4)
Supplement: Supplementary file 4 — Supplementary Information 4. [file 41598_2021_93048_MOESM4_ESM.docx]

import delimited C:\Users\Vanda\Desktop\Personal\Research\Thirst\thirst.csv

ttest age, by (ngt)

tab gender ngt, chi2 exact

tab race ngt, chi2 exact

tab htn ngt, chi2 exact

tab cancer ngt, chi2 exact

tab dm ngt, chi2 exact

tab oldcva ngt, chi2 exact

tab ihd ngt, chi2 exact

tab ckd ngt, chi2 exact

tab respi ngt, chi2 exact

tab depression ngt, chi2 exact

tab gicomplaints ngt, chi2 exact

tab infectionelsewhere ngt, chi2 exact

tab trauma ngt, chi2 exact

tab respiratory ngt, chi2 exact

tab cvs ngt, chi2 exact

tab urinary ngt, chi2 exact

tab neurology ngt, chi2 exact

tab hematological ngt, chi2 exact

tab metabolic ngt, chi2 exact

tab electivesurgery ngt, chi2 exact

tab headnecklumps ngt, chi2 exact

tab ivfluids ngt, chi2 exact

tab ppis ngt, chi2 exact

tab opioids ngt, chi2 exact

tab antihtn ngt, chi2 exact

tab corticosteroids ngt, chi2 exact

tab nsaids ngt, chi2 exact

tab diuretics ngt, chi2 exact

tab tca ngt, chi2 exact

tab anticholinergics ngt, chi2 exact

tab antipsychotics ngt, chi2 exact

ranksum howlonghaveyoubeeninhospitalford, by (ngt)

tab wereyouadmittedtoicuhd ngt, chi2 exact

ranksum durationofngtnbmhours, by (ngt)

tab wereyouonngtbefore ngt, chi2 exact

tab surgeryintervention ngt, chi2 exact

tab swallowingimpairment ngt, chi2 exact

tab poororalintake ngt, chi2 exact

tab thirst ngt, chi2 exact

ttest tdupdated, by (ngt)

ttest tiupdated, by (ngt)

ttest howmuchpainareyouin, by (ngt)

ttest howhungryareyou, by (ngt)

ttest age, by (thirst)

tab gender thirst, chi2 exact

tab race thirst, chi2 exact

tab htn thirst, chi2 exact

tab cancer thirst, chi2 exact

tab dm thirst, chi2 exact

tab oldcva thirst, chi2 exact

tab ihd thirst, chi2 exact

tab ckd thirst, chi2 exact

tab respi thirst, chi2 exact

tab depression thirst, chi2 exact

tab gicomplaints thirst, chi2 exact

tab infectionelsewhere thirst, chi2 exact

tab trauma thirst, chi2 exact

tab respiratory thirst, chi2 exact

tab cvs thirst, chi2 exact

tab urinary thirst, chi2 exact

tab neurology thirst, chi2 exact

tab hematological thirst, chi2 exact

tab metabolic thirst, chi2 exact

tab electivesurgery thirst, chi2 exact

tab headnecklumps thirst, chi2 exact

tab ivfluids thirst, chi2 exact

tab ppis thirst, chi2 exact

tab opioids thirst, chi2 exact

tab antihtn thirst, chi2 exact

tab corticosteroids thirst, chi2 exact

tab nsaids thirst, chi2 exact

tab diuretics thirst, chi2 exact

tab tca thirst, chi2 exact

tab anticholinergics thirst, chi2 exact

tab antipsychotics thirst, chi2 exact

ranksum howlonghaveyoubeeninhospitalford, by (thirst)

tab wereyouadmittedtoicuhd thirst, chi2 exact

tab ngt thirst, chi2 exact

ranksum durationofngtnbmhours, by (thirst)

tab wereyouonngtbefore thirst, chi2 exact

tab hyper thirst, chi2 exact

tab hypo thirst, chi2 exact

tab eu thirst, chi2 exact

tab didanydoctorornurseaskyouaboutth thirst, chi2 exact

tab didanydoctorornurseattempttotrea thirst, chi2 exact

ttest tdupdated, by (thirst)

ttest tiupdated, by (thirst)

ttest howmuchpainareyouin, by (thirst)

ttest howhungryareyou, by (thirst)
